# A Commentary on “PTX3 is an Extrinsic Oncosuppressor Regulating Complement-Dependent Inflammation in Cancer”

**DOI:** 10.3389/fonc.2015.00118

**Published:** 2015-05-28

**Authors:** Francesco Di Virgilio

**Affiliations:** ^1^Section of Pathology, Oncology and Experimental Biology, Department of Morphology, Surgery and Experimental Medicine, University of Ferrara, Ferrara, Italy

**Keywords:** cancer, inflammation, pentraxins, complement, tumor suppressor gene

Inflammation is a constant feature of tumors. The tumor microenvironment (TME) is rich in inflammatory cells and inflammatory mediators that strongly affect tumor growth and progression as well as metastatic spreading. It has been known for a while that tumor-infiltrating inflammatory cells such as myeloid-derived suppressor cells (MDSCs) and tumor-associated macrophages (TAM) promote tumor growth, and accordingly several soluble factors, such as the CCL2 chemokine, the pro-inflammatory cytokines IL-1, IL-6, and TNFα, the pro-angiogenic factor VEGF are enriched in the TME (1). Nevertheless, no oncogene or oncosuppressor genes have been so far found to encode molecules belonging to the humoral innate immune system.

A main constituent of the soluble arm of innate immunity are the pentraxins, a superfamily of highly conserved immune effector molecules comprised the short pentraxins (C reactive protein, CRP, and serum amyloid P, SAP, component) and the long pentraxin PTX3 (2). Pentraxins interact with complement and with complement-regulating factors to effect microbe recognition and disposal, with wide-range implications for host defense and regulation of inflammation. Short pentraxins are synthesized in the liver and released in the blood at the inception of inflammation. On the contrary, PTX3 is produced locally, not only by macrophages and neutrophils but also by endothelial and smooth muscle cells, in response to cytokines, pathogen-associated molecular patterns (PAMPs), intact microbes, oxidized-LDL, or HDL. PTX3 has been shown to play an important role in resistance against selected pathogens, and is under evaluation as an antimicrobic agent.

Bonavita et al. now show that PTX3 has a pivotal role in controlling tumor growth, and this is related to its ability to modulate the inflammatory response (3). Carcinogenesis was investigated in *Ptx3*^−/−^ and *Ptx3*^+^*^/^*^+^ mice treated with 3-methylcholantrene (3-MCA) or 7,12-dimethylbenz [α] anthracene/terephthalic acid (DMBA/TPA) to induce formation of sarcomas or papillomas/skin carcinomas, respectively. Twice as many *Ptx3*^−/−^ mice developed sarcomas compared to *Ptx3*^+^*^/^*^+^, and incidence and multiplicity of papillomas and progression to carcinomas were also much higher in *Ptx3*^−/−^than in *Ptx3*^+^*^/^*^+^. Interestingly, and in support of the prevalent origin of PTX3 from immune cells, the main source of PTX3 were infiltrating leukocytes and endothelial cells, but not tumor cells. Since PTX3 had no direct effects on tumor cell growth, attention was focused on cancer-related inflammation (CRI). Sarcomas growing in *Ptx3*^−/−^ host showed a much higher macrophage and neutrophil infiltrate, and higher levels of CCL2, CXCL2, TNFα, IL-6, IL-1β, and VEGF, suggesting an exacerbated tumor-associated inflammatory response. The crucial mechanistic link to dysregulated activation of inflammation in the *Ptx3*^−/−^ mice was shown to be complement activation. Both C3 and C5a were found to be deposited in higher amounts in *Ptx3*^−/−^ versus *Ptx3*^+^*^/^*^+^ homogenates, while the complement regulatory protein factor H was found significantly lower, suggesting that lack of PTX3 caused an unrestricted activation of complement-dependent inflammation as a consequence of defective factor H recruitment.

Inflammation is likely promoting cancer via multiple pathways, such as inhibition of anti-tumor cytotoxic responses, secretion of growth-promoting cytokines, release of angiogenic factors, but it may also favor tumor genetic instability. Bonavita et al. addressed this issue by investigating frequency of mutations in two genes, *Trp53* and *Kras*, that are target of several carcinogens, 3-MCA included (3). Quite interestingly, while frequency of *Kras* mutations was not increased, frequency of *Trp53* mutations was significantly higher in *Ptx3*^−/−^ versus *Ptx3*^+^*^/^*^+^ mice, pointing to a progressive loss of *Trp53*-dependent oncosuppressor function in the *Ptx3*^−/−^ host. Of relevance for human pathology, the *Ptx3* gene was found to be silenced by methylation in several human tumors.

Macrophages, MDSCs, several cytokines, and growth factors are thought to be essential components of the TME and have a prominent role in tumor progression (4), while complement was not included so far within the components of tumor-promoting inflammation. Now, the study by Bonavita et al. shows that complement should be considered a factor of cancer-promoting inflammation in its own right, and identifies the long pentraxin PTX3 as a crucial modulator of the complement-dependent tumor-promoting pathway (3). These observations, on one hand, underline the role of PTX3 as an extrinsic tumor suppressor gene, and on the other, give an additional, paradigmatic, demonstration of the fundamental role played by inflammation in tumorigenesis.

Chronic, low-grade inflammation is a constant feature of all malignant tumors, whether they are epidemiologically related to inflammation or not. CRI can be due to stimulation of the host inflammatory response by tumor cells, or tumor cell-derived soluble factors, or to specific activation of inflammatory pathways by oncogenes. These different pathways result in the production of a cellular and biochemical microenvironment, the TME, that is conducive to tumor progression and metastatic spreading. However, how and whether host genes encoding inflammatory mediators determine tumor progression is an as yet open question. In this context, identification of the PTX3 gene as a key player in cancer may also help to better rationalize the role of inflammation in tumor growth.

We should not be dogmatic in the interpretation of CRI. Inflammation may not always be “good” for the tumor and “bad” for the host. We know that tumor-infiltrating immune cells (e.g., γδ IL-17-producing T cells, CD8^+^ T lymphocytes, or type I macrophages) can kill tumor cells (5), and that high TNFα doses can inhibit tumor growth (6). Coley (7) showed as early as the late 1890s that bacterial extracts could be used to treat cancer, and in the same years, similar observations on the “anti-cancer activity of inflammatory exudates” were also reported by the Italian Pathologist Eugenio Centanni. Nowadays, urologists use bacillus Calmette–Guerin (BCG) for local treatment of bladder cancer, and with success! It is therefore clear that CRI can be “good” or “bad” for the host. How can we turn “bad” CRI into “good” CRI? It is likely that a switch might be located at the level of host inflammatory cells stimulation by PAMPs and/or damage-associated molecular patterns (DAMPs). The “Coley” “mélange” of bacterial extracts, like the BCG suspension used in bladder cancer, promotes a strong TLR-mediated response that facilitates development of protective adaptive immunity. This favorable outcome might not only be due to an efficient PAMP recognition but also to a large and concomitant release of DAMPs by injured tumor as well as host cells. It has been proposed that the “way” tumor cells die, also as a consequence of chemotherapy, is crucial for the development of an efficient adaptive immune response (immunogenic cell death), since factors released from dying cells (e.g., ATP, calreticulin, or high-mobility group box 1, HMGB1, protein) prime tumor-infiltrating dendritic cells for an efficient Ag presentation to CD8^+^ T lymphocytes. Stimulation of the P2X7 receptor, activation of the NLRP3 inflammasome and enhanced IL-1β release are components of this virtuous pathway. In addition, it should be noted that some experimental tumors have a stronger tendency than others to induce the generation of a “good” inflammatory microenvironment, protective for the host, probably because they are intrinsically and potently immunogenic. It is fair to say that molecular mechanisms that turn “good” into “bad” inflammation are not clear, yet every effort should be made to get a better insight into this crucial biological switch point because successful treatment of cancer patients might in the end reside in our ability to effectively modify CRI.

## Conflict of Interest Statement

The author declares that the research was conducted in the absence of any commercial or financial relationships that could be construed as a potential conflict of interest.
